# Multifaceted and Age-Dependent Phenotypes Associated With Biallelic *PNPLA6* Gene Variants: Eight Novel Cases and Review of the Literature

**DOI:** 10.3389/fneur.2021.793547

**Published:** 2022-01-06

**Authors:** Lorenzo Nanetti, Daniela Di Bella, Stefania Magri, Mario Fichera, Elisa Sarto, Anna Castaldo, Alessia Mongelli, Silvia Baratta, Silvia Fenu, Marco Moscatelli, Maria Teresa Bonati, Andrea Martinuzzi, Caterina Mariotti, Franco Taroni

**Affiliations:** ^1^Unit of Medical Genetics and Neurogenetics, Fondazione Istituto di Ricovero e Cura a Carattere Scientifico (IRCCS) Istituto Neurologico Carlo Besta, Milan, Italy; ^2^Unit of Rare Neurodegenerative and Neurometabolic Diseases, Fondazione Istituto di Ricovero e Cura a Carattere Scientifico (IRCCS) Istituto Neurologico Carlo Besta, Milan, Italy; ^3^Unit of Neuroradiology, Fondazione Istituto di Ricovero e Cura a Carattere Scientifico (IRCCS) Istituto Neurologico Carlo Besta, Milan, Italy; ^4^Unit of Medical Genetics, Institute for Maternal and Child Health Istituto di Ricovero e Cura a Carattere Scientifico (IRCCS) Burlo Garofalo, Trieste, Italy; ^5^Conegliano Research Center, Istituto di Ricovero e Cura a Carattere Scientifico (IRCCS) Eugenio Medea, Conegliano, Italy

**Keywords:** cerebellar ataxia, spastic paraplegia, hypogonadotropic hypogonadism, chorioretinal dystrophy, Gordon Holmes syndrome, Boucher Neuhauser syndrome, Oliver Mc Farlane syndrome

## Abstract

A wide spectrum of neurodegenerative diseases has been associated with pathogenic variants in the *PNPLA6* (patatin-like phospholipase domain-containing protein 6) gene, including spastic paraplegia type 39, Gordon—Holmes, Boucher—Neuhauser, Oliver—Mc Farlane, and Laurence—Moon syndromes. These syndromes present variable and overlapping clinical symptoms, encompassing cerebellar ataxia, hypogonadotropic hypogonadism, chorioretinal dystrophy, spastic paraplegia, muscle wasting, peripheral neuropathy, and cognitive impairment. In the present study, we performed a wide genetic screening in 292 patients presenting with ataxia or spastic paraplegia using a probe-based customized gene panel, covering >200 genes associated with spinocerebellar diseases. We identified six novel and four recurrent *PNPLA6* gene variants in eight patients (2.7%). Six patients presented an infantile or juvenile onset (age <18), and two patients had an adult onset. Cerebellar ataxia was observed in seven patients and spastic paraplegia in one patient. Progression of cerebellar symptoms was slow in all patients, who retained ambulation even after a mean disease duration of 15 years. Brain MRI showed cerebellar atrophy in 6/8 patients, more pronounced in superior and dorsal vermis lobules (I to VII). Additional clinical features included hypogonadotropic hypogonadism (5/8), growth hormone deficiency (2/8), peripheral axonal neuropathy (4/8), cognitive impairment (3/8), chorioretinal dystrophy (2/8), and bilateral vestibular areflexia with a reduced visual vestibule-ocular reflex (1/8). In accordance with previous studies, chorioretinal dystrophy was the most frequent presenting symptom in early onset patients, hypogonadotropic hypogonadism in juvenile onset cases, and cerebellar ataxia in adult patients. One patient had an initial clinical presentation compatible with Cerebellar Ataxia with Neuropathy and Vestibular Areflexia Syndrome (CANVAS), but no pathological expansions in the *RFC1* gene. In conclusion, patients with *PNPLA6* variants present a variable age of onset spanning from infancy to adulthood, and each clinical symptom has an age-dependent manifestation thus requiring a multi-systemic diagnostic approach. The description of patients presenting very late-onset cerebellar ataxia suggests that *PNPLA6* genetic screening should also be considered in the diagnostic workout of adult cerebellar ataxia.

## Introduction

Pathogenic variants in the *PNPLA6* gene(-encoding patatin-like phospholipase domain containing protein 6) have been demonstrated to cause a number of variable neurodegenerative diseases. This gene codes for the enzyme neuropathy target esterase (NTE), an endoplasmic reticulum-localized lysophospholipase that deacylates phosphatidylcholine and lysophosphatidylcholine, and has been studied for its involvement in the pathogenesis of organophosphorus compound-induced delayed neuropathy (1).

Pathogenic variants in the *PNPLA6* gene have been originally described in patients presenting a neurological phenotype characterized by early-onset spastic paraplegia, motor neuropathy, and distal muscle wasting. The disease was classified among the groups of the Hereditary Spastic Paraplegias as SPG type 39 (SPG39, MIM612020) (1–3).

Recently, bi-allelic *PNPLA6* gene variants have been detected in patients affected by two clinical syndromes described more than 50 years ago and named Gordon—Holmes (GH, MIM212840) and Boucher—Neuhauser (BN, MIM215470) (4). The typical features of the two clinical syndromes are cerebellar ataxia and hypogonadotropic hypogonadism (HH), and BN is distinguished from GH for the presence of chorioretinal dystrophy (5).

Genetic screening in large series of cases with neurodegenerative diseases further widened the clinical phenotype associated with *PNPLA6* gene variants, identifying patients with trichomegaly, chorioretinal atrophy, and multiple pituitary hormone deficiencies, including growth hormone (G-H), gonadotrophins, and thyroid-stimulating hormone (TSH). This particular phenotype was named Oliver—Mc Farlane syndrome (OMcF; MIM 275400) (6–9). Pathogenic variants in the *PNPLA6* gene were also demonstrated to cause the Laurence—Moon syndrome (LM, MIM 245800) that includes all the symptoms of the Oliver—Mc Farlane syndrome plus neurological symptoms, such as ataxia, spastic paraplegia, and neuropathy (6). Pure cerebellar ataxia and isolated congenital Leber amaurosis or retinitis were also described in single families (7, 10, 11).

It is not yet fully understood why pathogenic variants in the *PNPLA6* gene are associated with a wide spectrum of phenotypes. The NTE plays a critical role in phosphatidylcholine metabolism, membrane phospholipid trafficking, and maintenance of axonal integrity (12).

NTE enzymatic activity was demonstrated to be reduced in patient fibroblasts and mouse models (6, 13); however, a clear relationship between the variability in the NTE activity and the phenotypical variability in patients with *PNPLA6* gene variants has not been entirely established (6, 13).

In the present study, we describe the clinical phenotype of 8 Italian patients carrying novel and recurrent pathogenic *PNPLA6* gene variants and manifesting variable neurological and extra-neurological features.

## Methods

From 2015 to 2020, we investigated 292 index Italian patients recruited in our referral center for spinocerebellar diseases. The subjects were selected on the basis of the following criteria: (1) progressive ataxic syndrome or spastic paraplegia; (2) exclusion of infectious, autoimmune, or cerebrovascular diseases; (3) absence of pathogenic variants associated with spinocerebellar ataxia (SCA) types 1, 2, 3, 6, 7, and 17, Friedreich ataxia, and hereditary spastic paraplegia types 4 and 7 (SPG4; SPG7).

Genomic DNA was extracted from venous peripheral blood lymphocytes by standard procedures. NGS-targeted resequencing analysis was performed using a probe-based customized gene panel (Illumina Nextera Rapid Capture Custom Kit - Illumina Inc., San Diego, CA, USA) or an Agilent SureSelect QXT custom kit (Agilent Technologies, Santa Clara, CA, USA), including >200 genes associated with ataxia and spastic paraplegia (Supplementary Table 1).

Written informed consent was obtained from all the participants (or guardians of participants) in the study in accordance with the local Ethic Committee Board.

## Results

Eight out of the 292 index patients (2.7%) were demonstrated to carry homozygous or compound heterozygous *PNPLA6* pathogenic or likely pathogenic gene variants. Clinical and genetic characteristics of patients carrying *PNPLA6* gene variants are summarized in Table 1.

**Table 1:** Clinical and genetic characterization of eight patients with *PNPLA6* gene variants.

**Patient**	**1**	**2**	**3**	**4**	**5**	**6**	**7**	**8**
c.DNA change	**c.2891G>T**	**c.2504_2505del**	**c.597+1G>A**	**c.3816_3819del** c.3931C>T	c.2944_2947dup	**c.348dup**	c.3403C>T	c.2990C>T
	**c.3667C>T**	**c.3547C>T**	c.3403C>T		c.3931C>T	**c.348dup**	c.3403C>T	c.2990C>T
Protein change	**p.Gly964Val** **p.Arg1223Trp**	**p.Glu835AlafsTer19** p.Arg1135Trp ^10^	**p.?** p.Arg1135Trp^10^	**p.Cys1272Ter** p.Arg1311Trp^14^	p.Arg983GlnfsTer38^4^ p.Arg1311Trp^14^	**p.Pro117AlafsTer7p. Pro117AlafsTer7**	p.Arg1135Trp^10^ p.Arg1135Trp^10^	p.Ser997Leu^4^ p.Ser997Leu^4^
Gender	Male	Male	Female	Female	Male	Male	Female	Male
First symptom	Spastic gait	Blurred vision	Hypogonadism	Ataxic Gait	Short stature	Hypogonadism	Ataxic gait	Ataxic gait
Age at onset	3	6	14	9	10	18	47	55
Diagnosis	SPG	BN	BN	GH	GH	GH	Ataxia	Ataxia
Age at last exam	30	23	70	28	40	54	52	69
SARA score	n.a.	8	22	10	n.a.	n.a.	13.5	10
Nystagmus	+	–	+	+	+	+	+	+
Babinski sign	+	+	+	–	–	–	+	–
LL spasticity	+	+	–	+	–	–	+	–
Gait ataxia	–	+	+	+	+	+	+	+
Cognitive impairment	+	+	–	–	–	+	–	–
Neuropathy	+	+	–	–	–	–	+	+
Cerebellar atrophy	–	+	+	+	–	+	+	+
Small hypophysis	–	+	–	+	+	–	–	–
Retinal dystrophy	–	+	+	–	–	-	–	–
Hypogonadism	–	+	+	+	+	+	–	–
Other features	Distal weakness; scoliosis	Strabismus; GH deficiency	–	–	–	Severe osteoporosis	–	Vestibular areflexia

*Novel PNPLA6 variants are indicated with bold; LL, lower limb; SPG, spastic paraplegia; BN, Boucher Neuhauser; GH, Gordon Holmes. Superscript numbers refers to References 4; 10; 14*.

The patients carried four previously described pathogenic *PNPLA6* variants (4, 10, 14) and six novel variants. Three novel variants were truncating, two were missense, and one variant was predicted to affect a splice site (Table 1).

The novel missense variants c.2891G > T and c.3667C > T were *in trans* in Patient 1, and were absent (GnomAD-genome) or very rare (GnomAD-exome: T = 0.000008) in control population databases. According to ACMG criteria, both variants can be classified as Likely Pathogenic (Class 4) variants (15).

The splice site variant c.597 + 1G > A was *in trans* with the previously described c.3403C > T missense variant in Patient 3 (10), was absent in control population databases (GnomAD-exome and genome), predicted to “break/abolish the donor site” (Human Splicing Finder), and classified as “pathogenic” (Class 5 variant) (15).

Clinical presentations of our subjects spanned through the clinical spectrum of neurodegenerative diseases associated with *PNPLA6* gene variants: one patient manifested spastic paraplegia at age 3; one patient had chorioretinal dystrophy at age 6 and was subsequently diagnosed as having BN syndrome; one subject had chorioretinal dystrophy and HH at age 14 and was also diagnosed as BN; three patients had gait ataxia associated with HH or G-H deficiency (ages 9, 10, and 18) and were diagnosed as having GH syndrome; two subjects presented a late-onset cerebellar ataxia at ages 47 and 55 (Table 1).

Clinical diagnosis was highly dependent on the initial symptom of the patient and on the age at the onset. In fact, in infantile cases, the onset was mainly characterized by visual defects; in juvenile cases, the first clinical signs were associated with hormone deficiencies; and, in late-onset cases, the predominant presentation was characterized by cerebellar symptoms.

However, during the course of the disease, all the subjects developed common additional clinical symptoms: 7/8 patients manifested a progressive cerebellar syndrome, 5/8 hypogonadotropic hypogonadism, 4/8 peripheral axonal neuropathy and/or lower limb spasticity, 2/8 chorioretinal dystrophy, and 1 subject had bilateral vestibular areflexia with poor visual vestibule-ocular reflex (VVOR).

Cognitive impairment was reported in 3/8 patients: two subjects (Patients 1 and 2, Table 1) showed intellectual deficit at ages 5 and 6, in concomitance with other clinical manifestations of the disease. Both subjects necessitated dedicated learning support at school. In the third subject (Patient 8), mild cognitive impairment was disclosed in adulthood and ascertained by neurocognitive tests (ENB2, brief neuropsychological examination-2).

Cerebellar symptoms were the most recurrent clinical features, having a mean age of onset 31 (range 9–55), and very slow progression. All the patients were ambulatory at the last examination and had a mean SARA score of 12.7 (range 8–22) after mean disease duration of 15 years (range 2–30). Two index patients had a very late-onset cerebellar ataxia at age 47 (pt 7) and 55 (pt 8) and no additional *PNPLA6*-associated clinical features, in particular chorioretinal dystrophy or hypogonadotropic hypogonadism. Patient 7 had spastic ataxia complicated by peripheral motor neuropathy. The patient had a sibling aged 59, affected by cerebellar ataxia since age 51. No other clinical or genetic data are available as he refused any clinical evaluation.

Patient 8 had an initial clinical presentation compatible with Cerebellar Ataxia with Neuropathy and Vestibular Areflexia Syndrome (CANVAS), manifesting ataxia with cerebellar atrophy, axonal neuropathy, and vestibular areflexia. For this subject, the test for pathogenic repeat expansions in *RFC1* was performed in parallel with NGS analysis, and the result excluded the diagnosis of CANVAS.

**Figure 1:**
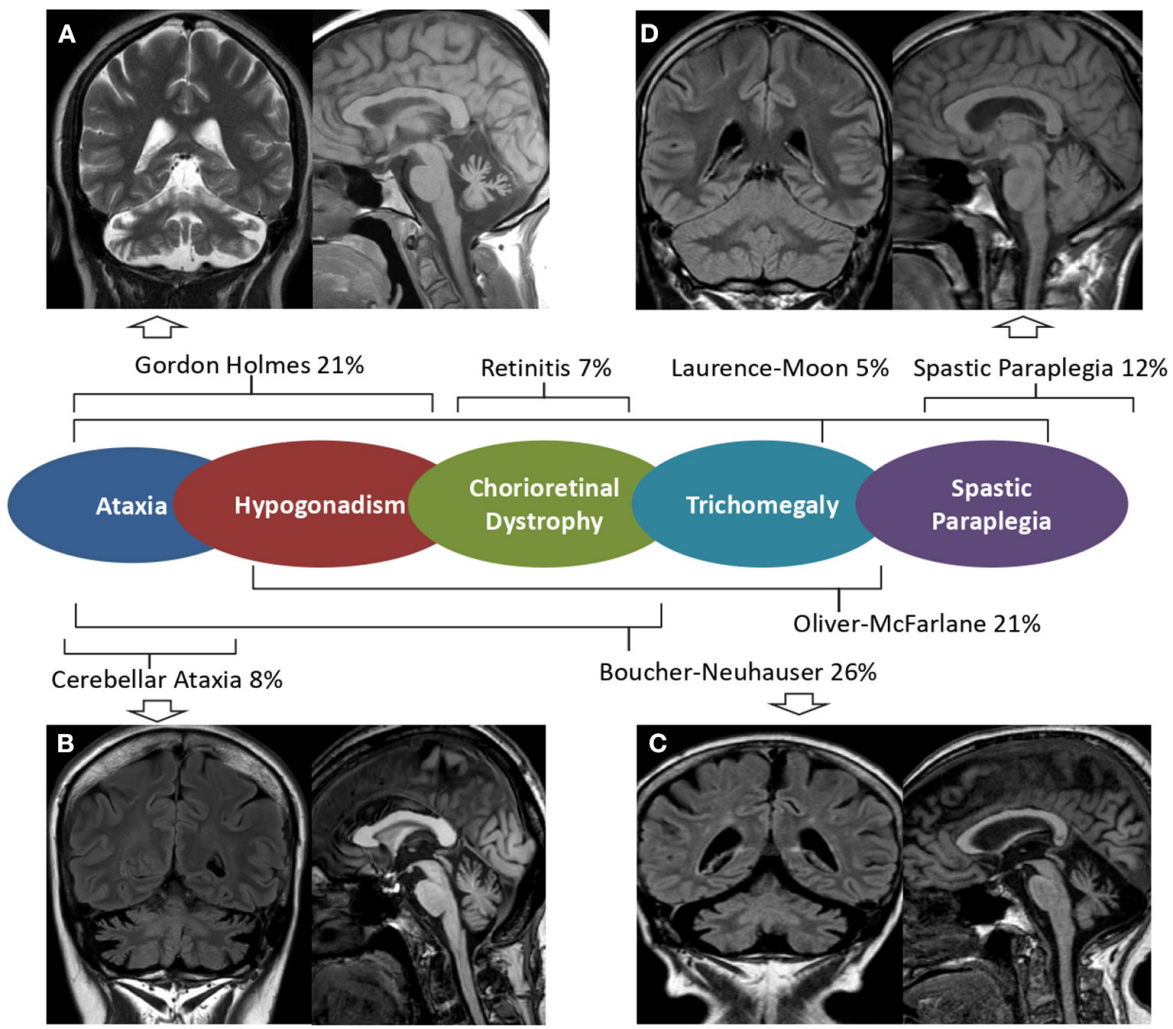
Brain MRI in *PNLA6*-associated phenotypes. Mid-sagittal T1 weighted and coronal FLAIR or T2 w brain MRI images of patients with *PNPLA6*-associated clinical phenotypes. Severe cerebellar atrophy and slightly hyperintense dentate nuclei are shown in patients with Gordon Holmes syndrome [**(A)**, Patient 4], late-onset cerebellar ataxia [**(B)**, Patient 7], and Boucher Neuhauser syndrome [**(C)**, Patient 3]. No MRI abnormalities are detectable in patients presenting Spastic Paraplegia [**(D)**, Patient 1]. A central scheme summarizes the frequency of the syndromic diagnoses reported in the literature.

Brain MRI scans demonstrated cerebellar atrophy in 6/7 ataxic patients with more pronounced atrophy in superior and dorsal vermal lobules (I to VII), compared to caudal lobules (VIII-X) (Figure 1).

Pituitary hormone deficiencies were also observed in patients with ataxia: hypogonadotropic hypogonadism in 5 out of 7 patients, and G-H deficiency in 2 patients. Brain MRI detected a reduced volume hypophysis in 5 out of 7 patients.

Patient 1 had a “not-ataxic” phenotype characterized by early-onset (age 3) progressive spastic paraplegia with cognitive impairment (QI = 49), and scoliosis (Table 1). At age 15, hand muscle weakness and amyotrophy were observed, and, in the subsequent years, lower limb distal muscles were affected, and progressive muscle wasting was detected. Nerve conduction studies revealed an axonal motor neuropathy more pronounced at median nerves and lower limb motor nerves. The disease progression was slow, and the patient was able to walk without support at the last examination (age 30). No cerebellar symptoms, hypogonadotropic hypogonadism, or retinal dystrophy were observed, and cerebellar atrophy was not detected at brain MRI (Figure 1).

## Discussion

Up to date, *PNPLA6* pathogenic gene variants were identified worldwide in 76 patients from 49 families. We revised the literature and summarized clinical (Table 2) and genetic (Supplementary Table 2) findings described in all reported *PNPLA6* patients.

**Table 2:** Clinical/genetic characterization of patients reported in the literature grouped for initial syndromic diagnosis.

**Diagnosis and** **Symptoms at onset**	**Fam/pt**	**Protein change** **(allele 1)**	**Protein change** **(allele 2)**	**Onset age** **(mean,years)**	**References**
Isolated retinitis	1/1	p.T311I	p.R983Qfs^*^38	1	(7)
	1/1	p.L366Sfs^*^28	p.T996A	4	(10)
	1/1	c.2068-10A>G	p.A1098T	14	(10)
Chorioretinopathy 100%	1/1	p.Q708^*^	p.A1098T	8	(10)
	1/1	p.R1135W	c.1697+3A>G	1	(10)
Oliver Mc Farlane and Laurence Moon syndromes	1/3	c.199-2A>T	p.D1077N	5	(7)
	1/1	p.L366Sfs^*^28	p.G1081R	1	(7)
	1/1	p.L476P	p.D1077N	2	(7)
	1/1	c.1829+2T>G	p.V1167A	1	(6)
	1/1	p.Q658^*^	p.G1081R	5	(7)
	1/4	p.G678R	p.R983Qfs^*^38	1	(6)
Chorioretinopathy 86% G-H deficiency 7% Hypogonadism 7%	1/1	p.R983Qfs^*^38	p.G1081R	1	(6)
	1/1	p.R983Qfs^*^38	p.G1081R	1	(6)
	1/1	p.S997L	c.3702+1G>A	7	(10)
	1/2	p.R1051Q	p.G1128S	6	(6)
	1/1	p.R1060W	p.G1081R	5	(7)
	1/1	p.V1167A	exon 14-20dup	1	(6)
	1/1	p.Q497H	p.G1123R	1	(9)
	1/1	p.S1159Y	p.S1159Y	12	(8)
Boucher Neuhauser syndrome	1/1	p.V215D	p.R1135Q	10	(16)
	1/1	p.L766P	p.W1129^*^	7	(17)
	1/1	p.E835Afs^*^19	p.R1135W	6	Present study
	1/4	p.T1010I	p.T1010I	2	(4)
Hypogonadism 43% Cerebellar ataxia 35% Chorioretinopathy 28%	1/1	p.S997L	p.P1074L	14	(4)
	1/1	c.597+1G>A	p.R1135W	14	Present study
	1/1	p.C429^*^	p.G964S	14	(18)
	1/1	p.S997L	p.S1125R	14	(19)
	1/1	p.G1081V	p.W1130C	14	(20)
	1/3	p.W1130C	p.W1130C	21	(21)
	1/1	p.T927A	p.S1127_G1128insVS	5	(21)
	1/1	p.L935Rfs^*^86	p.R1311Q	11	(22)
	1/2	p.R983Qfs^*^38	p.R1314G	6	(4)
	1/1	p.S1127C	p.S1127C	5	(23)
Gordon Holmes syndrome	1/1	p.P117Afs^*^7	p.P117Afs^*^7	18	Present study
	1/1	c.199-2A>T	p.R1311W	13	(14)
	1/2	p.D376Gfs^*^18	p.R1099C	1	(13)
Hypogonadism 69% Cerebellar ataxia 31%	1/1	p.G530W	p.F1018S	14	(4)
	1/1	p.Q707P	p.H1082T	12	(24)
	1/4	p.E754Q	p.R1313^*^	14	(25)
	1/2	p.G832Valfs^*^27	p.R1311W	1	(13)
	1/1	p.R983Qfs^*^38	p.R1311W	10	Present study
	1/2	p.S1127C	p.S1127C	1	(13)
	1/1	p.Y48^*^	p.R241G	3	(14)
	1/2	c.2068-1G>C	p.V1062M	9	(4)
	1/1	p.R1313^*^	p.D1077N	23	(17)
	1/1	p.C1272^*^	p.R1311W	9	Present study
SPG39	1/3	p.P1297S	p.A1064T	3	(26)
	1/1	p.R558^*^	exon 17-18 del	6	(3)
Spastic paraplegia100%	1/1	p.R890H	p.R983Qfs^*^38	5	(1)
	1/2	p.M1012V	p.M1012V	2	(1)
	1/1	p.G964V	p.R1223W	3	Present study
	1/1	p.R983Qfs^*^38	p.V1052G	20	(4)
Cerebellar ataxia	1/1	p.V215I	p.G792E	4	(4)
	1/1	p.R266W	p.R266W	1	(27)
Cerebellar ataxia 100%	1/1	p.S997L	p.S997L	56	Present study
	1/1	p.R1135W	p.R1135W	47	Present study
	1/2	p.V1235M^#^ p.D1262V^#^	p.V1235M^#^ p.D1262V^#^	12	(11)

# *In cys PNPLA6 gene variants.*

Asterisk (^*^) indicates stop codon.

**Figure 2:**
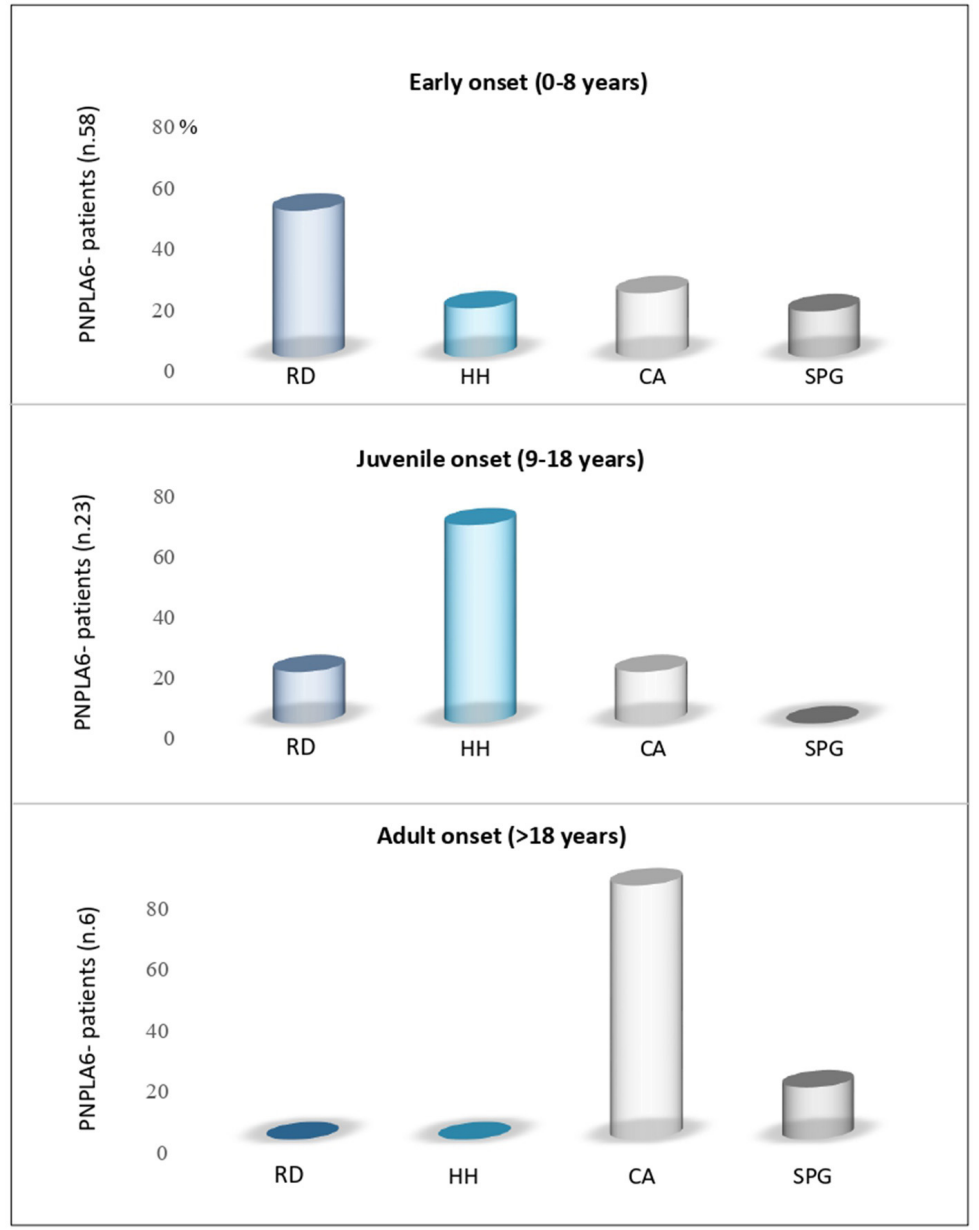
Age-dependent *PNPLA6*-associated clinical symptoms. Review of the literature for manifesting symptoms reported in patients with *PNPLA6* variants: Subjects with an early onset had predominantly retinal dystrophy (RD); juvenile cases had hypogonadotropic hypogonadism (HH) as presenting symptom, and adult cases had cerebellar ataxia (CA) or spastic paraplegia (SPG).

The age at the onset was highly variable, with the majority of patients (70%) presenting an infantile onset, ranging from 0 to 8 years (53/76 patients). Juvenile-onset was reported in 27% of cases (range, 9–18 years, 19/76 patients), and only 6% of cases manifested the disease in adulthood (4/76 patients) (Figure 2).

The presenting phenotype seems to be age-dependent. In fact, chorioretinal dystrophy was the most frequent clinical presentation in infantile cases (51%), hypogonadotropic hypogonadism in juvenile-onset cases (68%), and cerebellar ataxia in late-onset cases (75%) (Figure 2).

Initial clinical diagnosis reported in the literature was very heterogeneous and probably influenced by the large number of syndromes described in the last decades before the identification of the *PNPLA6*-associated phenotypes.

In fact, the syndromic diagnosis in patients reported in the literature was BN in 26% of patients, GH in 21%, Oliver—Mc Farlane Syndrome in 21%, Hereditary Spastic Paraplegia in 12%, cerebellar ataxia in 8%, and Laurence—Moon Syndrome 5%. In addition, four patients had a diagnosis of isolated retinitis (5%), and a single case was diagnosed as having Leber congenital amaurosis (Table 2).

**Figure 3:**
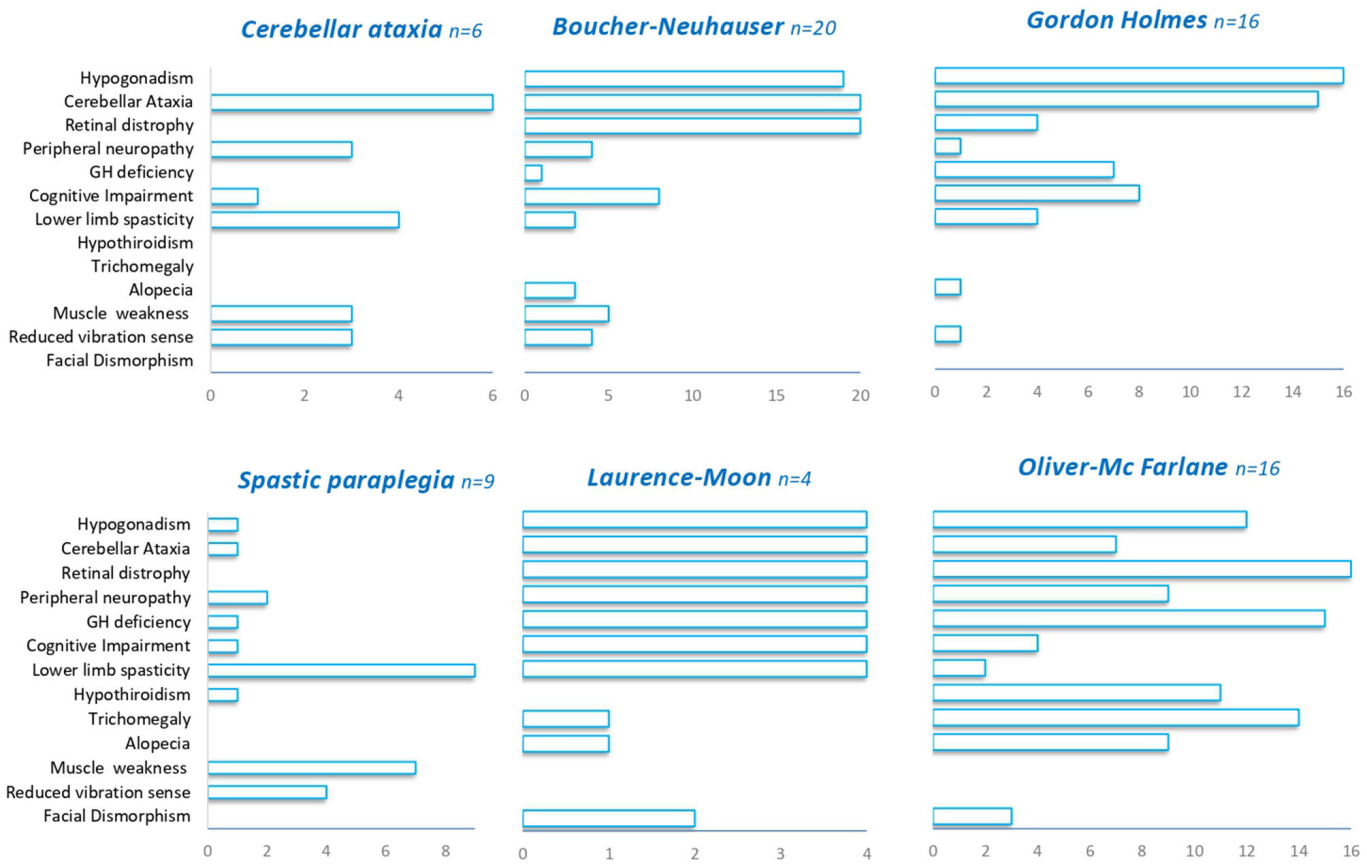
Clinical features in *PNPLA6*-associated phenotypes. A summary of the different clinical features (Y-axis) observed in *PNPLA6*-associated phenotypes.

Irrespectively of the clinical presentation at diagnosis, the clinical features observed during the course of the diseases largely overlap in most of the patients with *PNPLA6* variants. Hypogonadotropic hypogonadism was found in 74% of cases and has been detected in all *PNPLA6*-associated phenotypes except in SPG39. Cerebellar ataxia was described in 68% of the patients encompassing all the syndromes associated with *PNPLA6*. Chorioretinal dystrophy was present in 65% of cases, being always observed in BN, LM, and OMF syndromes, and nearly absent in GH, spastic paraplegia, and late-onset cerebellar ataxia phenotypes (Figure 3). Late-onset cases have a pure spinocerebellar phenotype and do not manifest visual or endocrinological deficits.

In our series, we screened patients with either juvenile or an adult onset; we, therefore, observed hypogonadotropic hypogonadism and cerebellar ataxia as predominant presenting symptoms (88 and 63% of patients), while chorioretinal dystrophy was observed only in two patients (25%) (Table 1). Brain MRI confirmed that cerebellar atrophy is more pronounced in the superior and dorsal lobules than in caudal lobules (Figure 1). We also describe two unrelated patients with very late-onset cerebellar ataxia at age 47 (pt 7) and 55 (pt 8), and no chorioretinal dystrophy or hypogonadotropic hypogonadism (Table 1). Patient 8 had an initial clinical presentation compatible with CANVAS that was never described in patients with *PNPLA6* gene variants. Notably, poor VVOR suppression was previously described in two patients diagnosed as having BN. Deik et al. described a Spanish-Italian woman presenting amenorrhea during adolescence, followed by gait ataxia, retinal dystrophy, axonal neuropathy, and poor VVOR suppression manifested in the sixth decade (19). In addition, Tarnutzer et al. reported the case of a man presenting cerebellar ataxia, hypogonadotropic hypogonadism, retinal dystrophy, and poor VVOR suppression in the third decade (14).

Regarding the genetic variants identified in our series and in previous *PNPLA6* cases, we confirmed that pathogenic variants are identified across all the gene regions, although the majority are identified in the Phospholipid Esterase Domain (EST) (Supplementary Table 2).

Not only different variants were associated with different presenting phenotypes, but even the same recurrent *PNPLA6* gene variant can be found in subjects with different clinical manifestations.

For example, the most frequent variant worldwide, the c.2944_2947dupAGCC, has been previously identified in several cases, including our Patient 5. The patients presented different syndromes, including BN (4), GH (pt 5), Spastic Paraplegia (4), Oliver—Mc Farlane syndrome (6), Laurence—Moon syndrome (6), and Leber congenital amaurosis (7). The variability of clinical *PNPLA6*-phenotypes was further confirmed for the c.3403C > T (p.Arg1135Trp) variant. This *PNPLA6* variant is very frequent in our patients (4/16 alleles, 25%), and it was previously identified in a Chinese patient affected by BN (10).

It has to be noted, however, that recurrent variants were variably associated *in trans* with different pathogenic *PNPLA6* gene variants, thus suggesting a different and probably interrelating contribution of both *PNPLA6* alleles to the final disease phenotype.

It has been demonstrated that *PNPLA6* knock-out mice are embryonic lethal, and a pathogenic variant creating an early stop codon in human *PNPLA6* has only been identified in compound heterozygous patients (28).

Truncating variants represent 18% (13/72) of all *PNPLA6* gene variants, and homozygous patients always carried missense variants, localized both in the EST domain and in Cyclic Nucleotide Binding-Homology (CNB) regions (28).

**Figure 4:**
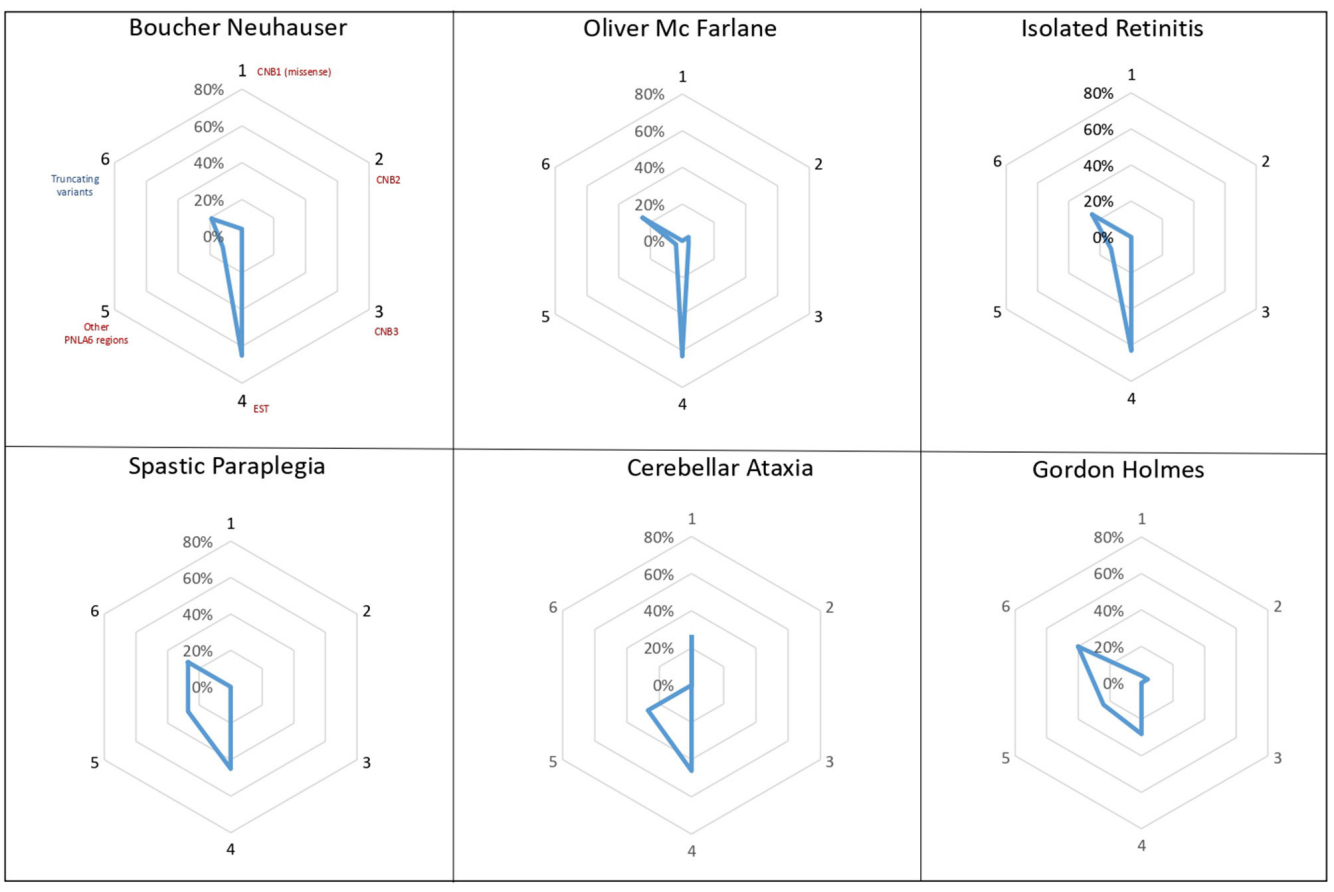
*PNPLA6* gene variants. Diagrams illustrate the frequency of *PNPLA6* gene variant types in patients described in the literature. Missense variants (in red) have been grouped according to their domain localization: the Cyclic Nucleotide Binding-Homology (CNB) domains (1-2-3), the Phospholipid Esterase (EST) domain (4), or other PNPLA6 regions (5). Truncating variants (in blue) have been reported as a unique group, irrespectively to their localization within the *PNPLA6* gene.

Previous studies suggested that the localization of *PNPLA6* variants could impact the development of different clinical phenotypes, with certain variants toward N terminal, causing preferentially ataxia and spastic paraplegia, while variants toward C terminal cause neurological symptoms with hypogonadism (26). Analyzing the different distributions and types of *PNPLA6* variants within the protein domains, we observed that missense variants in the EST domain are more frequent in phenotypes associated with chorioretinal dystrophy (63–65%) (Figure 4). Truncating variants have the same frequency in all phenotypes (25–40%), except for cerebellar ataxia in which truncating variants were not described. However, it remains extremely challenging to provide more accurate genotype-phenotype information and a precise correlation with the NTE residual enzymatic activity.

In conclusion, *PNPLA6* gene variants cause a wide spectrum of clinical syndromes and a great variability in the age of onset spanning from early infancy to adulthood. The complexity of the diagnosis and clinical management of the patients requires the collaboration of several specialists depending on the age of the patient and the specific clinical features (pediatrician, ophthalmologist, endocrinologist, geneticist, and adult neurologist).

The clinical presentation greatly influences the diagnostic workout. For example, early-onset cases are often evaluated by ophthalmologists and initially screened for genes associated with visual impairment. Juvenile- and adult-onset cases are likely to be evaluated by different physicians depending on the specific presenting symptom (29–31). We suggest that *PNPLA6* gene screening should also be considered in the diagnostic workout of patients with late-onset cerebellar signs, including patients presenting a CANVAS-like phenotype, when pathological expansions in the *RFC1* gene are not detected.

## Data Availability Statement

Anonymized data which is not published within this article will be made available by request from any qualified investigator. The data presented in this study are deposited in Zenodo repository (https://zenodo.org), accession number 10.5281/zenodo.5793958. Requests to access these datasets should be directed to ufficioricerca@istituto-besta.it.

## Ethics Statement

The studies involving human participants were reviewed and approved by Fondazione IRCCS Istituto Neurologico Carlo Besta, Milan, Italy. The patients/participants provided their written informed consent to participate in this study. Written informed consent was obtained from the individual(s) for the publication of any potentially identifiable images or data included in this article.

## Author Contributions

LN, DD, SM, CM, and FT: study design, conception, execution, writing of the draft, and review manuscript. MF, ES, AC, AMo, SF, MM, SB, MB, and AMa: data collection and writing of the draft. All authors contributed to the article and approved the submitted version.

## Funding

This work was partly funded by grants CP 20/2018 (Care4NeuroRare) from the Fondazione Regionale per la Ricerca Biomedica (FRRB) and RF-2018-12367768 from the Italian Ministry of Health to FT.

## Conflict of Interest

The authors declare that the research was conducted in the absence of any commercial or financial relationships that could be construed as a potential conflict of interest.

## Publisher's Note

All claims expressed in this article are solely those of the authors and do not necessarily represent those of their affiliated organizations, or those of the publisher, the editors and the reviewers. Any product that may be evaluated in this article, or claim that may be made by its manufacturer, is not guaranteed or endorsed by the publisher.
