# Supplementary material for: Multifaceted and Age-Dependent Phenotypes Associated With Biallelic PNPLA6 Gene Variants: Eight Novel Cases and Review of the Literature
Source: Front Neurol. 2022 Jan 6;12:793547. doi: 10.3389/fneur.2021.793547 (PMC8770815; doi:10.3389/fneur.2021.793547)
Supplement: Supplementary file 1 [file Table_1.DOCX]

**Supplementary Table 1.** Ataxia-HSP NGS gene panel.

| **Gene** | **>20X target region (%, mean ±SD)** | **Disease** |
| --- | --- | --- |
| *AAAS* | 99.6 ± 0.7 | AAAS (AR) |
| *ABCB7* | 100 | ASAT (XL) |
| *ABCD1* | 100 | ALD (XL) |
| *ABHD12* | 98.5 ± 2 | Refsum disease / PHARC (AR) |
| *ADCK3* | 100 | SCAR9 (AR) |
| *AFG3L2* | 99.6 ± 0.7 | SCA28 (AD), SPAX5 (AR) |
| *ALDH18A1* | 100 | SPG9A (AD) |
| *ALS2* | 100 | IAHSP (AR) |
| *AMACR* | 100 | AMACRD (AR) |
| *AMPD2* | 100 | SPG63 (AR) |
| *ANG* | 100 | ALS9 (AD) |
| *ANO10* | 84 ± 2.9 | SCAR10 (AR) |
| *AP4B1* | 100 | SPG47 (AR) |
| *AP4E1* | 100 | SPG51 (AR) |
| *AP4M1* | 100 | SPG50 (AR) |
| *AP4S1* | 99.5 ± 1.1 | SPG52 (AR) |
| *AP5Z1* | 100 | SPG48 (AR) |
| *APTX* | 100 | AOA1 (AR) |
| *ARHGEF28* | 97.9 ± 0 | SLA (AD) |
| *ARL6IP1* | 100 | SPG61 (AR) |
| *ARSA* | 100 | MLD (AR) |
| *ARSI* | 99.8 ± 0.5 | SPG66 (AR) |
| *ASAH1* | 100 | SMAPME (AR) |
| *ATCAY* | 98.5 ± 2.8 | ATCAY (AR) |
| *ATL1* | 100 | SPG3A/HSN1D (AD) |
| *ATM* | 100 | ATM (AR) |
| *ATP13A2* | 98.7 ± 1.6 | PARK9 (AR) |
| *ATP1A2* | 99.9 ± 0.2 | FHM2/AHC1 (AD) |
| *ATP1A3* | 99.5 ± 1.2 | DYT12/ AHC2/CAPOS (AD) |
| *ATP2B3* | 99.8 ± 0.5 | SCAX1 (XL) |
| *ATP2B4* | 100 | ATP2B4 (AD) |
| *ATP7A* | 100 | Menkes disease (XL), SMAX3 (XL) |
| *ATP7B* | 100 | Wilson disease (AR) |
| *ATR* | 97.7 ± 0 | SCKL1 (AR) |
| *B4GALNT1* | 78.7 ± 3.2 | SPG26 (AR) |
| *BICD2* | 100 | SMALED2 (AD) |
| *BSCL2* | 100 | SPG17 /HMN5C (AD) |
| *C10ORF2* | 100 | PEOA3 (AD), PRLTS5/MTDPS7 (AR) |
| *C12ORF65* | 99.9 ± 0.2 | SPG55 (AR) |
| *C19ORF12* | 100 | SPG43 (AR) |
| *CACNA1A* | 99.4 ± 0.8 | EA2/FHM 1/EIEE42/SCA6 (AD) |
| *CACNA1G* | 100 | SCA42 (AD) |
| *CACNB4* | 100 | EA5 (AD) |
| *CAMTA1* | 98 ± 0.4 | CANPMR (AD) |
| *CCDC88C* | 100 | SCA40 (AD) |
| *CCT5* | 97.9 ± 1.8 | CCT5 (AR) |
| *CHMP2B* | 100 | ALS17 (AD) |
| *CLN3* | 100 | CLN3 (AR) |
| *CLN5* | 100 | CLN5 (AR) |
| *CLN6* | 100 | CLN6 (AR) |
| *CLN8* | 100 | CLN8 (AR) |
| *COASY* | 100 | NBIA6 (AR) |
| *COQ2* | 90.4 ± 3.4 | COQ10D1 (AR) |
| *COQ4* | 100 | COQ10D7 (AR) |
| *COQ6* | 100 | COQ10D6 (AR) |
| *COQ9* | 99.9 ± 0.3 | COQ10D5 (AR) |
| *CP* | 100 | Aceruloplasminemia (AR) |
| *CPT1C* | 100 | SPG73 (AD) |
| *CSTB* | 100 | EPM1A (AR) |
| *CTSD* | 98.1 ± 3.5 | CLN10 (AR) |
| *CTSF* | 97.4 ± 4.2 | CLN13 (AR) |
| *CWF19L1* | 100 | SCAR17 (AR) |
| *CYP27A1* | 100 | CTX (AR) |
| *CYP2U1* | 98.6 ± 2 | SPG56 (AR) |
| *CYP7B1* | 100 | SPG5 (AR) |
| *DARS* | 100 | HBSL (AR) |
| *DARS2* | 100 | LBSL (AR) |
| *DCAF17* | 99.9 ± 0.2 | Woodhouse-Sakati (AR) |
| *DCTN1* | 100 | HMN7B (AD) |
| *DDHD1* | 100 | SPG28 8AR) |
| *DDHD2* | 100 | SPG54 (AR) |
| *DNAJC19* | 100 | MGCA5 (AR) |
| *DNAJC3* | 100 | ACPHD (AR) |
| *DNAJC5* | 100 | CLN4B (AD) |
| *DNM2* | 99.9 ± 0.3 | CMT2M / CMTDIB /CMTDI1 (AD) |
| *DNMT1* | 99.8 ± 0.5 | ADCADN (AD), HSN1E (AD) |
| *EEF2* | 100 | SCA26 (AD) |
| *ELOVL4* | 100 | SCA34 (AD) |
| *ELOVL5* | 100 | SCA38 (AD) |
| *ENTPD1* | 100 | SPG64 (AD) |
| *ERLIN1* | 100 | SPG62 (AR) |
| *ERLIN2* | 100 | SPG18 (AR) |
| *FA2H* | 99.6 ± 0.6 | SPG35 (AR) |
| *FAM134B* | 96.8 ± 3.6 | HSAN2B (AR) |
| *FGF14* | 100 | SCA27 (AD) |
| *FIG4* | 100 | ALS11 (AD); CMT4J/BTOP/YVS (AR) |
| *FLRT1* | 100 | SPG68 (AR) |
| *FLVCR1* | 100.1 | AXPC1 (AR) |
| *FMR1* | 100 | FMR1 (XLD) |
| *FTL* | 100 | NBIA3 (AR) |
| *FXN* | 99.6 ± 0.7 | FRDA (AR) |
| *GAD1* | 100 | CPSQ1 (AR) |
| *GALC* | 99.5 ± 1 | KRABBE (AR) |
| *GAN* | 100 | GAN1/ARCMT2 (AR) |
| *GARS* | 100 | CMT2D / HMN5A (AD) |
| *GBA* | 100 | Gaucher disease, (AR) |
| *GBA2* | 100 | SPG46 (AR) |
| *GBE1* | 100 | GSD4 (AR), APBN (AR) |
| *GCH1* | 97.4 ± 4.1 | DYT5 (AD/AR), HPABH4B (AR) |
| *GJB1* | 100 | CMTX1 (XL) |
| *GJC2* | 99 ± 0.5 | SPG44 (AR), HLD2 (AR) |
| *GLB1* | 100 | GM1 (AR) |
| *GOSR2* | 100 | EPM6 (AR) |
| *GRID2* | 100 | SCAR18 (AR) |
| *GRM1* | 100 | SCAR13 (AR) |
| *GRN* | 100 | CLN11 (AR) |
| *HEXA* | 93 ± 0 | TAY-SACHS (AR) |
| *HEXB* | 100 | Sandhoff disease (AR) |
| *HNRNPA1* | 100 | ALS20 (AD) |
| *HNRNPA2B1* | 100 | IBMPFD2(AD) |
| *HSPD1* | 100 | SPG13 (AD), HLD4 (AD, AR) |
| *HTRA2* | 99.4 ± 1.4 | MGCA8 (AR) |
| *IBA57* | 98.2 ± 2.8 | MMDS3 (AR), SPG74 (AR) |
| *IFRD1* | 100 | SCA18 (AD) |
| *ITPR1* | 99.8 ± 0.4 | SCA15/SCA19 (AD) |
| *KCNA1* | 100 | EA1/EAM (AD) |
| *KCNA2* | 100 | EIEE32 (AD) |
| *KCNC3* | 85 ± 3 | SCA13 (AD) |
| *KCND3* | 100 | SCA19 (AD) |
| *KCNJ10* | 100 | SESAME (AR) |
| *KIAA0196* | 100 | SPG8 (AD) |
| *KIAA0226* | 100 | SCAR15 (AR) |
| *KIF1A* | 99.3 ± 1.2 | SPG30 (AD, AR), MRD9 (AD), HSN2C (AR) |
| *KIF1C* | 100 | SPAX2 (AR) |
| *KIF5A* | 100 | SPG10 (AD), NEIMY (AD), ALS25 (AD) |
| *L1CAM* | 100 | MASA (XL), SPG1 (XL) |
| *LYST* | 100 | LYST (AR) |
| *MARS* | 100 | SPG70 (AR), CMT2U (AD) |
| *MARS2* | 100.2 | SPAX3 (AR) |
| *MED25* | 92.4 ± 1 | ARCMT2B/CMT2B2 (AR) |
| *MFN2* | 100 | CMT2A2/HMSN6 (AD) |
| *MFSD8* | 100 | CLN7 (AR) |
| *MMACHC* | 100 | GDHS (AR) |
| *MPZ* | 100 | CMT2J / CMT1B / CMTDID (AD) |
| *MRE11A* | 92.6 ± 0 | ATLD1 (AR) |
| *MTPAP* | 100 | SPAX4 (AR) |
| *MTTP* | 94.9 ± 0 | ABL (AR) |
| *NEFL* | 100 | CMT2E / CMT1F (AD) |
| *NFU1* | 100 | MMDS1 (AR) |
| *NIPA1* | 94.2 ± 2.7 | SPG6 (AD) |
| *NPC1* | 96.6 ± 1.3 | NPC1 (AR) |
| *NPC2* | 100 | NPC2 (AR) |
| *NT5C2* | 100 | SPG65 (AR) |
| *OPA1* | 100 | OPA1 (AD) |
| *OPA3* | 98.5 ± 2.9 | OPA3 (AD) |
| *PANK2* | 99.5 ± 1.1 | NBIA1 (AR) |
| *PAX6* | 100 | PAX6 (AD) |
| *PCNA* | 100 | ATM-L (AR) |
| *PDSS1* | 88.9 ± 2 | COQ10D2 (AR) |
| *PDSS2* | 100 | COQ10D3 (AR) |
| *PDYN* | 100 | SCA23 (AD) |
| *PEX10* | 87.3 ± 1.7 | PBD6A (AR) |
| *PEX6* | 93.2 ± 1.5 | PBD4B (AR) |
| *PEX7* | 91.9 ± 3.5 | PBD9B (AR) |
| *PGAP1* | 96.3 ± 0.3 | SPG67 (AR) |
| *PHKA1* | 100 | GSD9D (XKP |
| *PHYH* | 99.4 ± 1.3 | REFSUM (AR) |
| *PIK3R5* | 100 | AOA3 (AR) |
| *PLA2G6* | 99.2 ± 1.6 | INAD1/NBIA2B (AR) |
| *PLEKHG4* | 100 | SCA31 (AD) |
| *PLP1* | 100 | PMD/SPG2 (XL) |
| *PNKP* | 91.3 ± 0 | AOA4/MCSZ/EIEE10 (AR) |
| *PNPLA6* | 100 | BNHS (AR), SPG39 (AR) |
| *POLG* | 100 | MTDPS4A/MTDPS4B/SANDO/PEOB1 (AR), PEOA1 (AD) |
| *PPT1* | 100 | CLN1 (AR) |
| *PRICKLE1* | 100 | EPM1B (AR) |
| *PRKCG* | 100 | SCA14 (AD |
| *PRNP* | 100 | GSS/CJD/HDL1/FFI (AD) |
| *PRRT2* | 100 | ICCA, EKD1, BFIS2 (AD) |
| *PRX* | 98.7 ± 2.4 | CMT4F (AR) |
| *RAB3GAP2* | 100 | WARBM2 (AR) |
| *REEP1* | 91.6 ± 3.1 | HMN5B/SPG31 (AD) |
| *REEP2* | 100 | SPG72 (AD, AR) |
| *RNF170* | 100 | SNAX1 (AD) |
| *RNF216* | 99.9 ± 0.3 | GDHS (AR) |
| *RTN2* | 100 | SPG12 (AD) |
| *SACS* | 100 | ARSACS (AR) |
| *SCARB2* | 100 | EPM4 (AR) |
| *SCN1A* | 100 | FHM3/GEFSP2/EIEE6 (AD) |
| *SCN8A* | 100.1 | EIEE13/BFIS5/CIAT (AD) |
| *SETX* | 97.5 ± 0 | AOA2/SCAR1 (AR), ALS4 (AD) |
| *SIL1* | 100 | MSS (AR) |
| *SLC16A2* | 99.9 ± 0.2 | SPG22 (XL), AHDS (XL) |
| *SLC1A3* | 100 | EA6 (AD) |
| *SLC1A4* | 100 | SPATCCM (AR) |
| *SLC25A46* | 96.9 ± 0 | HMSN6B (AR) |
| *SLC33A1* | 100 | CCHLND (AR), SPG42 (AD) |
| *SLC52A2* | 99.9 ± 0.5 | BVVLS2 (AR) |
| *SNX14* | 100 | SCAR20 (AR) |
| *SOD1* | 100 | ALS1 (AD) |
| *SPAST* | 100 | SPG4 (AD) |
| *SPG11* | 100 | CMT2X/SPG11 (AR) |
| *SPG20* | 100 | SPG20 (AR) |
| *SPG21* | 100 | SPG21 (AR) |
| *SPG7* | 99.3 ± 1.3 | SPG7 (AR) |
| *SPTAN1* | 100 | EIEE5 (AD) |
| *SPTBN2* | 100 | SCA5 (AD), SCAR14 (AR) |
| *STUB1* | 100 | SCAR16 (AR) |
| *SYNE1* | 99.4 ± 0 | SCAR8 (AR) |
| *SYT14* | 100 | SCAR11 (AR) |
| *TDP1* | 100 | SCAN1 (AR) |
| *TECPR2* | 100 | SPG49 (AR) |
| *TFG* | 100 | SPG57 (AR), HMSNO (AD) |
| *TGM6* | 99.9 ± 0.2 | SCA35 (AD) |
| *TH* | 98.1 ± 3.9 | SEGAWA (AR) |
| *TMEM240* | 99.9 ± 0.2 | SCA21 (AD) |
| *TPP1* | 100 | CLN2 (AR), SCAR7 (AR) |
| *TRPC3* | 99.6 ± 0.6 | SCA41 (AD) |
| *TTBK2* | 100 | SCA11 (AD) |
| *TTC19* | 87.7 ± 1.4 | MC3DN2 (AR) |
| *TTPA* | 99.4 ± 0.9 | AVED (AR) |
| *USP8* | 100 | SPG59 (AR) |
| *VAMP1* | 100 | SPAX1 (AD) |
| *VCP* | 99.6 ± 0.8 | ALS14/IBMPFD1/CMT2Y (AD) |
| *VPS13A* | 100 | CHAC (AR) |
| *VPS37A* | 100 | SPG53 (AR) |
| *VWA3B* | 100 | SCAR22 (AR) |
| *WDR45* | 100 | NBIA5 (XLD) |
| *WDR48* | 100 | SPG60 (AR) |
| *WFS1* | 100 | Wolfram disease (AR) |
| *WWOX* | 100 | SCAR12/EIEE28 (AR) |
| *ZFR* | 100 | SPG71 (AR) |
| *ZFYVE26* | 100 | SPG15 (AR) |
| *ZFYVE27* | 100 | SPG33 (AD) |
| *ZNF592* | 100 | SCAR5 (AR) |

AD, autosomal dominant; AR, autosomal recessive; XL, X-linked
