# Supplementary material for: Multifaceted and Age-Dependent Phenotypes Associated With Biallelic PNPLA6 Gene Variants: Eight Novel Cases and Review of the Literature
Source: Front Neurol. 2022 Jan 6;12:793547. doi: 10.3389/fneur.2021.793547 (PMC8770815; doi:10.3389/fneur.2021.793547)
Supplement: Supplementary file 2 [file Table_2.DOCX]

| **Supplementary table 2.** *PNPLA6* genetic variants identified in patients with variable phenotypes | | | | | | | |
| --- | --- | --- | --- | --- | --- | --- | --- |
| **cDNA change**  **NM_006702.5** | **Protein change** | **Predicted effect** | **Protein domain** | **N. Alleles** | **N. Pts** | **Phenotype** | **Ref.** |
| c.144T>G | p.Y48* | truncating | - | 1 | 1 | GH | [14] |
| c.199-2A>T | p.? | splice-site | - | 4 | 4 | GH, OF | [7,14] |
| c.644T>A | p.V215D | missense | CNB1 | 1 | 1 | BN | [20] |
| c.643G>A | p.V215I | missense | CNB1 | 1 | 1 | CA | [4] |
| c.721C>G | p.R241G | missense | CNB1 | 1 | 1 | GH | [14] |
| c.796C>T | p.R266W | missense | CNB1 | 2 | 1 | CA | [21] |
| c.932C>T | p.T311I | missense | - | 1 | 1 | LCA | [7] |
| c.1126dupG | p.D376Gfs*18 | truncating | - | 2 | 2 | GH | [13] |
| c.1094dupC | p.L366Sfs*28 | truncating | - | 2 | 2 | OF, IR | [7, 10] |
| c.1287T>A | p.C429* | truncating | - | 1 | 1 | BN | [18] |
| c.1491G>T | p.Q497H | missense | CNB2 | 1 | 1 | OF | [9] |
| c.1427T>C | p.L476P | missense | CNB2 | 1 | 1 | OF | [7] |
| c.1672_1674delCGGinsTA | p.R558* | truncating | CNB2 | 1 | 1 | SP | [3] |
| c.1588G>T | p.G530W | missense | CNB2 | 1 | 1 | GH | [4] |
| c.1697+3A>G | p.? | splice-site | CNB2 | 1 | 1 | IR | [10] |
| c.1829+2T>G | p.? | splice-site | CNB3 | 1 | 1 | OF | [6] |
| c.1972C>T | p.Q658* | truncating | CNB3 | 1 | 1 | OF | [7] |
| c.2032G>C | p.G678R | missense | CNB3 | 4 | 4 | LM | [6] |
| c.2068-1G>C | p.? | splice-site | CNB3 | 3 | 3 | GH, IR | [4, 10] |
| c.2068-10A>G | p.? | splice-site | CNB3 | 1 | 1 | IR | [10] |
| c.2120A>C | p.Q707P | missense | - | 1 | 1 | GH | [24] |
| c.2122C>T | p.Q708* | truncating | - | 1 | 1 | IR | [10] |
| c.2669G>A | p.R890H | missense | - | 1 | 1 | SP | [1] |
| c.2297T>C | p.L766P | missense | - | 1 | 1 | BN | [26] |
| c.2260G>C | p.E754Q | missense | - | 4 | 4 | GH | [25] |
| c.2494_2495insTGTGGGCCTGGGG | p.G832Vfs*27 | truncating | - | 2 | 2 | GH | [13] |
| c.2375G>A | p.G792E | missense | - | 1 | 1 | CA | [4] |
| c.2890G>A | p.G964S | missense | - | 1 | 1 | BN | [18] |
| c.2779A>G | p.T927A | missense | - | 1 | 1 | BN | [22] |
| c.2800_2803dup | L935Rfs*86 | truncating | EST | 1 | 1 | BN | [23] |
| c.2944_2947dupAGCC | p.R983Qfs*38 | truncating | EST | 14 | 14 | OF, LM, BN, SP, LCA | [1, 4, 6, 7, 13] |
| c.3034A>G | p.M1012V | missense | EST | 4 | 2 | SP | [1] |
| c.2986A>G | p.T996A | missense | EST | 1 | 1 | IR | [10] |
| c.2990C>T | p.S997L | missense | EST | 3 | 3 | BN, OF | [4, 10] |
| c.3029C>T | p.T1010I | missense | EST | 8 | 4 | BN | [4] |
| c.3190G>A | p.A1064T | missense | EST | 3 | 3 | SP | [16] |
| c.3053T>C | p.F1018S | missense | EST | 1 | 1 | GH | [4] |
| c.3152G>A | p.R1051Q | missense | EST | 2 | 2 | OF | [6] |
| c.3295C>T | p.R1099C | missense | EST | 2 | 2 | GH | [13] |
| c.3155T>G | p.V1052G | missense | EST | 1 | 1 | SP | [4] |
| c.3178C>T | p.R1060W | missense | EST | 1 | 1 | OF | [7] |
| c.3184G>A | p.V1062M | missense | EST | 2 | 2 | GH | [4] |
| c.3221C>T | p.P1074L | missense | EST | 1 | 1 | BN | [4] |
| c.3367G>A | p.G1123R | missense | - | 1 | 1 | OF | [9] |
| c.3229G>A | p.D1077N | missense | - | 4 | 4 | GH, OF | [7, 26] |
| c.3380C>G | p.S1127C | missense | - | 4 | 2 | GH | [13] |
| c.3241G>C | p.G1081R | missense | - | 5 | 5 | OF | [6, 7] |
| c.3242G>T | p.G1081V | missense | - | 1 | 1 | BN | [27] |
| c.3387G>A | p.W1129* | truncating | - | 1 | 1 | BN | [26] |
| c.3244C>T | p.H1082Y | missense | - | 1 | 1 | GH | [24] |
| c.3404G>A | p.R1135Q | missense | - | 1 | 1 | BN | [20] |
| c.3292G>A | p.A1098T | missense | - | 2 | 2 | IR | [10] |
| c.3375C>G | p.S1125R | missense | - | 1 | 1 | BN | [17] |
| c.3377_3382dupTGTCCG | p.S1127_G1128insVS | in frame insertion | - | 1 | 1 | BN | [22] |
| c.3380C>G | p.S1127C | missense | - | 2 | 1 | BN | [19] |
| c.3382G>A | p.G1128S | missense | - | 2 | 2 | OF | [6] |
| c.3390G>C | p.W1130C | missense | - | 7 | 4 | BN | [22, 27] |
| c.3403C>T | p.R1135W | missense | - | 1 | 1 | IR | [10] |
| c.3476C>A | p.S1159Y | missense | - | 2 | 1 | OF | [8] |
| c.3500T>C | p.V1167A | missense | - | 2 | 2 | OF | [6] |
| c.3702+1G>A | p.? | splice site | - | 1 | 1 | OF | [10] |
| c.3703G>A | p.V1235M | missense | - | 2 | 2 | CA | [11] |
| c.3889C>T | p.P1297S | missense | - | 3 | 3 | SP | [16] |
| c.3785A>T | p.D1262V | missense | - | 2 | 2 | CA | [11] |
| c.3931C>T | p.R1311W | missense | - | 2 | 2 | GH | [13-14] |
| c.3932G>A | p.R1311Q | missense | - | 2 | 2 | BN | [23] |
| c.3937C>T | p.R1313* | truncating | - | 5 | 5 | GH | [25-26] |
| c.3940C>G | p.R1314G | missense | - | 2 | 2 | BN | [4] |
| exon 14–20 dup | p.? | intragenic duplication | - | 1 | 1 | OF | [6] |
| exon 17-18 del | p.? | intragenic deletion | - | 1 | 1 | SP | [3] |

Pts= Patients; GH=Gordon Holmes; BN= Boucher Neuhauser; CA= Cerebellar Ataxia; SP= Spastic Paraplegia; OF= Oliver-McFarlane; LM= Laurence-Moon; LCA= Leber congenital amaurosis; IR= isolated retinitis; Ref= reference; Protein domains: Cyclic Nucleotide Binding-Homology (CNB) domains (1-2-3), the Phospholipid Esterase (EST) domain.
